# Social Sharing of Emotions and Communal Appraisal as Mediators Between the Intensity of Trauma and Social Well-Being in People Affected by the 27F, 2010 Earthquake in the Biobío Region, Chile

**DOI:** 10.3389/fpsyg.2021.641793

**Published:** 2021-03-25

**Authors:** Carlos Reyes-Valenzuela, Loreto Villagrán, Carolina Alzugaray, Félix Cova, Jaime Méndez

**Affiliations:** ^1^Programa Andino de Derechos Humanos, Universidad Andina Simón Bolívar, Quito, Ecuador; ^2^Departamento de Psicología, Facultad de Ciencias Sociales, Universidad de Concepción, Concepción, Chile; ^3^Escuela de Psicología, Facultad de Ciencias Sociales, Universidad Santo Tomás, Santiago, Chile

**Keywords:** earthquake, trauma, social sharing of emotions, community appraisals, social well-being

## Abstract

The psychosocial impacts of natural disasters are associated with the triggering of negative and positive responses in the affected population; also, such effects are expressed in an individual and collective sphere. This can be seen in several reactions and behaviors that can vary from the development of individual disorders to impacts on interpersonal relationships, cohesion, communication, and participation of the affected communities, among others. The present work addressed the psychosocial impacts of the consequences of natural disasters considering individual effects via the impact of trauma and community effects, through the perception of social well-being, the valuation of the community and the social exchange of emotions. The aim of this study was to assess the relationship between individual reactions (i.e., intensity of trauma) and the evaluation of social and collective circumstances (i.e., social well-being) after the earthquake of 27F 2010 in Chile, through collective-type intervention variables not used in previous studies (i.e., social sharing of emotions and community appraisal). For this purpose, a descriptive, *ex post* facto correlational and cross-sectional methodology was carried on, with the participation of 487 people affected by the 2010 earthquake, 331 women (68%) and 156 men (32%), between 18 and 58 years old (*M* = 21.09; *SD* = 5.45), from the provinces of Ñuble and Biobío, VIII region, Chile. The measurement was carried out 4 years after the earthquake and the results show that greater individual than collective involvements were found, mainly in the coastal zone of the region. The mediation analysis showed that the relationship between the intensity of the trauma and social well-being occurs through a route that considers social sharing of emotions and community appraisal. These results indicate that the overcoming of individual affectations to achieve social well-being occurs when in the immediate post-disaster phases the affected communities activate shared emotional and cognitive processes, which allow them to jointly face subsequent threats and abrupt changes.

## Introduction

From the vulnerability approach (Cardona, 2001; Caviedes, 2015) the Latin American context, and specifically the Chilean context, presents an accumulation of weaknesses that can be grouped into different physical, economic, social or educational dimensions, which go beyond its geographical conditions (Ayala and Olcina, 2002; Blaikie et al., 2006; Rojas and Martínez, 2012). Romero and Romero (2015) identify that the effects of natural disasters depend largely on political-ecological factors that lead to their repercussions not being experienced in the same way throughout society, with the most vulnerable and socially excluded segments being the most affected, economically and territorially. Hewitt (1996) illustrates how the social structure constitutes a determining factor when considering the consequences of a natural disaster, pointing out that, in the evidence found, the distribution and amount of human losses in natural disasters is directly related to the socioeconomic condition of the affected people.

In recent decades, the condition of vulnerability in Chile has been aggravated by factors such as rapid urban growth and environmental damage, which has led to a decrease in the quality of life of the population, devastation of the country’s natural resources, a deterioration of natural landscapes and an impact on the national culture (Cardona, 2001; Instituto Nacional de Derechos Humanos, 2018).

Traditionally, the study of the consequences of natural disasters was from the clinical setting, fundamentally, from the evidence of impacts such as the diagnosis of post-traumatic stress disorder (PTSD) (Ayala and de Paúl Ochotorena, 2004; Friedman et al., 2007; Zhang et al., 2018) which does not consider mental health as a state of well-being that explores the perception of individuals of their own capacities and their potential contribution to the community (World Health Organization, 2013). This vision of trauma and disasters has had several detractors (Creamer, 2000; Cova et al., 2011; Crespo and Gómez, 2012) who question the definition of what constitutes a traumatic event (Brewin et al., 2009; Crespo and Gómez, 2012) because the clinical criteria presents a restrictive perspective on the impacts when it comes to accounting for more complex phenomena such as political violence or disasters which, in other words, refers to the lack of consideration of the context in these events (Blanco et al., 2016; Bennett et al., 2019). This has led to an overestimation of the diagnosis of PTSD and the assessment of the population in need for clinical intervention, along with an underestimation of the broader impact that these events present in communities (Bonanno et al., 2010). Moreover, the effects or consequences of disasters would be related to the social scenario in which they arise (Cova and Rincón, 2010).

In the face of these limitations, two key elements emerge to understand the effects of disasters studies: first, disasters can also elicit positive responses or effects, being a source of new learning or personal growth (Tedeschi and Calhoun, 1996; Bonanno, 2004; Vera et al., 2006) and second, that disasters cause impacts on communities, characterized by a weakened access to social support, impairment in interpersonal relationships and a decrease in the sense of community (Bonanno et al., 2010), disarticulating the social fabric, cohesion and participation, while affecting the leadership of community actors (Barrales et al., 2013).

Leiva-Bianchi et al. (2018) conducted a meta-analysis of the psychosocial impact of disasters considering war and catastrophe events, which precisely considered the effects on the individual, family and social environment of the populations affected by an event. These authors conclude that it is possible to understand the impact of these events through two independent axes: one, related to the “negative effects” (post-traumatic stress, stress, depression, anxiety, substance abuse, hate responses, disruptive behavior, and general psychopathology) and, the other one related to the “positive effects” (coping, post-traumatic growth, social support, emotional well-being and positive emotions). A second axis is related to “protection versus exposure” in which it is identified that at the end of protection there would be a maximum of resources such as social support or community participation to cope with the events, while at the end of exposure to the event these resources would be minimal or non-existent.

A relevant aspect is that traumas have a social nature, that they arise and are maintained in a context (Martín-Baró, 2003) in which, according to Blanco et al. (2016) it is of interest to place oneself in the previous situations (pre-traumatic situation), in the immediate impacts on people (destruction of beliefs about oneself) and in the community impacts (community destruction). Complementarily, there are studies that explain looting behaviors such as those identified in the 2010 Chile earthquake, based on the country’s pre-disaster conditions like social inequality and the predominance of a consumer society (Grandón et al., 2014). Other studies included the one where Mandavia and Bonanno (2018) investigate the effects of environmental stressors, identifying that disasters such as those produced by hurricanes and a subsequent economic crisis will generate a high impact on the mental health of the exposed community. Accordingly, one way to prepare communities and reduce their vulnerability to disasters is constituted by strategies that promote community participation and the prevention of the loss of psychosocial resources (Smith and Freedy, 2000). These strategies constitute an essential element in reducing levels of social vulnerability and would enable communities to access social and governmental resources to cope with these events. The evidence in natural disasters confirms that people who perceive social support and present a willingness to community organization have greater social cohesion, lower indicators of individual disorders (Figueroa et al., 2010), better indicators of well-being and post-traumatic growth (Villagrán et al., 2014; Wlodarczyk et al., 2016a,b).

Complementary to these findings, the approach to positive and adaptive aspects in individuals and communities implies considering the study of well-being as an essential component of health, as proposed by the World Health Organization (2003). Blanco and Díaz (2005) state that well-being includes social needs, problems and collective aspirations, in addition to support and social relationships which, in turn, are associated with the set of resources derived from the network of interpersonal relationships in which an individual and his community are inserted (Putnam, 2000; Keyes and Shapiro, 2004). In this regard, Keyes (1998) proposes the notion of social well-being that integrates social and cultural elements that promote mental health, in which social contact, interpersonal relationships, roots, community contacts and social participation would increase well-being in the communities. On the one hand, relationships have been found between social well-being and variables of different kinds such as life satisfaction, psychological well-being, resilience or personality styles (Zubieta and Delfino, 2010; Alzugaray et al., 2018). On the other hand, social well-being is associated with a sense of community (Tempier et al., 2012), specifically, where this sense of community would act as a predictor of social well-being (Albanesi et al., 2007). Kaniasty (2012) in a longitudinal study 1 year after a flood, found that participation in altruistic communities was associated with better interpersonal and community relationships and, conversely, dissatisfaction in such relationships predicted lower levels of well-being. Therefore, exposure to disasters mobilizes different individual and collective resources that contribute to later well-being, as found in the 2010 Chile earthquake (García et al., 2014; Villagrán et al., 2014; Wlodarczyk et al., 2016a,b, 2017).

In the Chilean context, the study of disasters has increased since the 2010 earthquake, and has combined research on clinical and community variables. In the first case, several studies evaluated PTSD, either in children and adolescents (Rincón et al., 2014), just in adolescents (Díaz et al., 2012) and in adults (Leiva-Bianchi, 2011). From the studies cited, it should be noted that, although PTSD was indeed found between 6 and 30%, the highest percentage of the population did not meet criteria for mental health pathology in the reviewed contexts. In contrast, other studies in Chile have considered the negative and positive community impacts of the earthquake: a qualitative research on people living in temporary camps found an increase in the sense of belonging in those affected who rebuilt their homes in the same place, however, they also reported increases in the levels of conflict and violence in the community (Grandón et al., 2016). In addition, Rojas et al. (2014) evaluated coastal communities in the Biobío region affected by the 2010 earthquake and tsunami, finding a decrease in household income, increased insecurity of the population and a slow reconstruction process. Evidence of well-being in the context of the 2010 earthquake has been found to be associated with communal growth and adaptive coping strategies (Leiva-Bianchi et al., 2012; Villagrán et al., 2014; Wlodarczyk et al., 2016b, 2017).

In this regard, early results have addressed the communal aspects that arise in disaster-affected communities, specifically through communal coping. This coping occurs when one or more individuals perceive a stressor as “our problem” and activate a shared or collaborative coping process: this involves a shared assessment of stress and a shared action orientation to manage stress in a group or community (Lyons et al., 1998). Thus, coping has been found to occur when there is a shared collective experience, which also involves a shared assessment of the stressful event, as well as a shared communication about stress and, finally, a mobilization in people to act collectively (Lyons et al., 1998; Leprince et al., 2018; Rentscher, 2019). In this way, a shared process is emphasized that leads to a communal confrontation, which makes possible better possibilities to deal with the impacts of the natural disaster (Villagrán et al., 2014; Wlodarczyk et al., 2016a,b; García and Wlodarczyk, 2018). In this context of shared coping, two key concepts emerge to understand how it occurs: the communal appraisal associated with the shared valuation of the experience of stress, which has been addressed in the studies by Lazarus and Folkman, and the social sharing of emotions raised by Bernard Rimé.

The notion of appraisal identifies a cognitive process in which an individual explores and assesses whether and how his environment is favorable to his or her well-being (Lazarus, 1966; Folkman et al., 1986). In particular, the appraisal presents a preponderant role in people’s evaluation before experiencing emotions and implies an adaptation to changing situations of the environment, which represents a subjective evaluation of the goals, objectives and the capacity of coping (Schmidt et al., 2010).

In this sense, it is of interest when the situations of the environment affect a group of people and the assessment they can make as a whole, especially when dealing with high intensity events that challenge individual resources (Villagrán et al., 2014). Although there are studies that have raised the notion of reappraisal as a communal coping strategy (Wlodarczyk et al., 2016a,b), here we consider communal appraisal as the entire collective evaluation that is made of the emotions in the face of events that equally affect a group. Hence, through the communal appraisal, the cognitive work of assessment is prevented from being solely individual, which could be directed to less adaptive forms, such as rumination (Weiss and Berger, 2010), which could impact on people’s basic beliefs (Janoff-Bulman, 1992), or be oriented toward cognitive avoidance behaviors or desires for a magical change in the problem (Villagrán et al., 2014).

On the other hand, the social sharing of emotions has been identified as the translation of an emotional experience into a socially shared language (Rimé et al., 1998; Rimé, 2009), which would allow the reconstruction of people’s beliefs through the transmission of shared feelings and which promotes the construction of a collective emotional atmosphere (Páez et al., 2007). At this point, living an emotional experience implies the need to share it with others, especially one of high intensity (Martínez Sánchez, 2011). In a disaster context, this has been identified more as a coping phase (Páez et al., 2001), rather than as an exchange that promotes wider social effects related to social structure, social norms or interpersonal relationships, among others (Rimé et al., 2020).

## Study Approach

This study addresses the context of the earthquake and tsunami of February 27, 2010 (27F) in Chile, which generated multiple social, cultural, political and economic impacts at the country level (CEPAL, 2010). Many individuals and families, especially in coastal areas, lost family members and material goods, along with the emergence of a sense of vulnerability, lack of protection and insecurity, because of the measures and actions of the Chilean State that presented deficiencies in relation to the basic organization of the supply of essential inputs and products to address the emergency and provide public safety (Letelier, 2010). Based on this, it is interesting to see how, after an initial stage of alarm at a later stage, people generate shared actions oriented at seeking meaning and understanding (Zech and Rimé, 2005) through verbal interaction that provokes a common evaluation of events (Von Scheve and Ismer, 2013), through the social sharing of emotions and community assessment. Although there is evidence from studies on the psychosocial impacts of 27F (e.g., García et al., 2016; Leiva-Bianchi et al., 2018), it has not been explored whether a shared assessment favored better levels of adaptation and social well-being.

Another aspect of interest relates to exploring the persistent individual and collective affectations in the context of threats and abrupt changes after the earthquake, given that a characteristic of the 27F earthquake was the scarce institutional preparation to face critical situations. Specifically, difficulties arose in territorial security, in the information provided by the authorities that was not clear, and in the limitations in the supply of basic services such as food, water and electricity (Torres-Méndez et al., 2018). Thus, it is expected that the inhabitants of the coastal area of the Biobío region (i.e., Concepción) will be more intensely affected, and will also be exposed to other phenomena such as the tsunami, in comparison to inland areas (i.e., Ñuble).

Thus, the purpose is to evaluate the relationship between individual reactions (i.e., intensity of the trauma) and the assessment of social circumstances after an earthquake (i.e., social well-being) through collective-type intervening variables not used in previous studies in 27F, such as the social sharing of emotions and the communal appraisal. This aim will provide further insight into the conditions immediately following a disaster and their possible impact on a positive evaluation of social functioning.

## Methodology

### Participants

The methodology used corresponded to a descriptive, *ex post* facto correlational and cross-sectional research design. The participants corresponded to 491 people affected by the 2010 earthquake, 331 women (68%) and 156 men (32%), between 18 and 58 years old (*M* = 21.09; *SD* = 5.45), from the provinces of Ñuble (32.4%) Concepción (63.3%) and Arauco (4.3%), Biobío region, Chile. The measurements were applied 4 years after the earthquake.

### Instruments

#### Trauma Intensity (IT) (Wlodarczyk et al., 2016a)

The emotional impact of the traumatic episode was assessed using three items: (A) “In general, how stressful or traumatic was the earthquake situation for you?” (B) “How intense was the experience for you?” (C) “To what extent did it cause you anxiety?” The response scale ranged from 1 (not at all) to 7 (a lot). A single factor was obtained through an exploratory factor analysis (EFA). The reliability of this scale was excellent (*a* = 0.87).

#### Communal Appraisal (CA)

The communal evaluation of emotions in the face of the earthquake was evaluated through five items: (a) “Did the group perceive that their discomfort decreased?” (b) “Did the group understand the situation of displacement?” (c) “Did the group control or resolve the situations?” (d) “Did the group manage relationships?” (e) “Did the group maintain or improve its image?” The response scale ranged from 1 (not at all) to 7 (a lot). A single factor was obtained by an EFA. Reliability for the scale was α **=** 0.82.

#### Social Sharing of Emotions (SSE) (Rimé, 2005)

It was evaluated by four questions about the frequency and the need to talk about the collective traumatic situations and impacts associated with the earthquake. E.g., “How frequently have you spoken about the events of 27F during the last week?” “How frequently have you heard people talk about the events of 27F during the last week?” The response options range from 1 (Nothing) to 4 (Much). The reliability of the scale was α = 0.81.

#### Social Well-Being

Social Well-Being Scale (SWB) (Keyes, 1998, adapted by Blanco and Díaz, 2004). A total of 15 items from the short Spanish version of Social Well-Being Scale (Bobowik et al., 2015) composed by five dimensions: social integration (e.g., You feel like you’re an important part of your community), social acceptance (e.g., “You believe that people are kind”), social contribution (e.g., “You think you have something valuable to give to the world”), social actualization (e.g., “You see society as continually evolving”), and social coherence (e.g., “The world is too complex for you”). The response range is from 1 (totally disagree) to 5 (totally agree). The reliability obtained for this scale was α = 0.88.

### Procedure and Data Analysis

This work was developed ensuring the fulfillment of the ethical principles of investigation on a national and international level. The participants were contacted directly, the purpose of the study was explained to them, the anonymous and confidential nature of their responses, and the possibility of withdrawing from the study at any time if they so wished. The participants were given the contacts of the primary care health centers (Centros de Salud Familiar, CESFAM) in the areas where they lived, where mental health staff contacts were available. This was in case they needed psychological counseling after participating in the study. Two versions of the questionnaire were created: an online version in which the questions were sent via e-mail to the participants, who answered voluntarily for 2 months. The paper version was answered mainly by the participants from the province of Ñuble. In both cases, the questionnaire consisted, first, of an informed consent and then contained a section where they had to fill in the socio-demographic data. Then came the items referring to the earthquake situation and the subsequent reactions, thoughts and actions, both individually and collectively. Subsequently, participants were asked to answer about trauma intensity, CA, SSE, and SBW.

A mediational analysis was carried out, using the macro PROCESS for SPSS, specifically model 6 (Hayes and Preacher, 2013), to contrast that social sharing of emotions and communal appraisal are mediating factors between the intensity of trauma and social well-being. In order to estimate the mediation model, a number of 10,000 bootstrap samples.

## Results

### Frequency of Losses and Level of Personal and Community Involvement

In relation to the frequency of various losses associated with the earthquake, human losses (13.1%), family crisis (9.9%), and economic losses (57.6%) were found. An index of individual affectation was constructed in relation to different impacts (e.g., changes in work activity, perception of dangers, changes in income) and an index of communal affectation (e.g., material damage in the neighborhood, presence of looting behaviors in the neighborhood, among others), which showed a low, medium and high level of affectation. Significant differences are evidenced in a greater affectation in women than in men, both in the individual affectation (χ^2^ = 8.01, *p* < 0.05) and in the communal one (χ^2^ = 5.59, *p* = 0.061). There are also significant differences in communal involvement by zones, being greater in the province of Concepción compared to Ñuble (χ^2^ = 42.23, *p* < 0.01).

### Correlates of the Individual and Communal Affectation With the Variables Under Study

To observe the relationship between individual and communal affectation with the variables under study, the correlations established a significant relationship between TI, CA, and SES with individual and communal involvement. However, there was no significant relationship between SWB and individual and collective affectation, but rather with some of its dimensions, specifically, acceptance, contribution and social coherence (see Table 1).

**TABLE 1:** Correlations between TI, SSE, CA and SBW with personal and community involvement.

Variables under study	Personal involvement	Community involvement
TI	0.64**	0.30**
SSE	0.35**	0.20**
CA	0.13*	0.09*
SBW	0.08	0.07
Social integration	0.11*	0.059
Social acceptance	0.10*	0.15**
Social contribution	0.12**	0.10*
Social actualization	0.08	0.02
Social coherence	−0.15**	−0.10*

*p < 0.05; **p < 0.01.

### Association Between Individual-Collective Involvement and SWB

**FIGURE 1:**
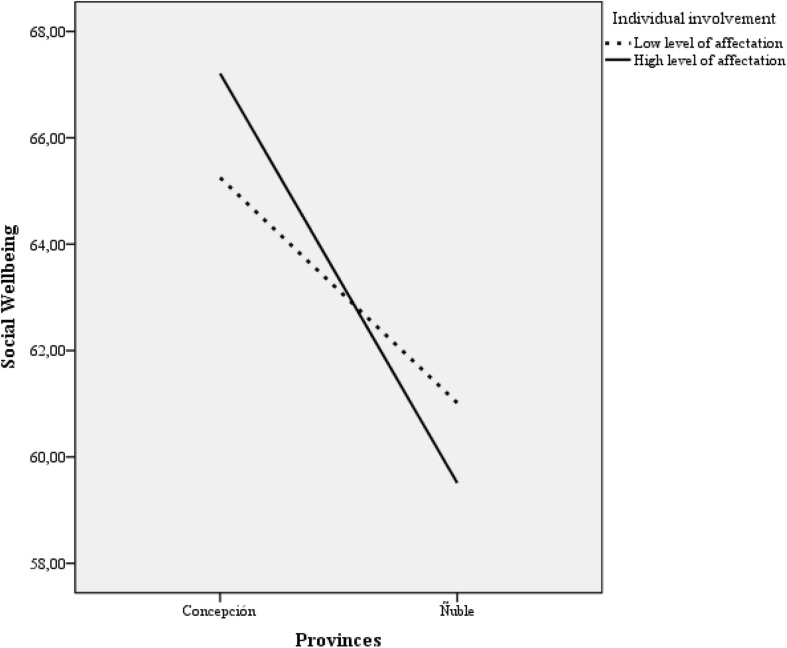
SBW by low and high level of affectation and province.

The association between individual and collective affectation according to the province of origin (i.e., Concepción and Ñuble) with social well-being was examined, considering that the province of Concepción also experienced a tsunami. The participants were divided by the median into high versus low individual and collective affectation. An ANOVA of 2 (province of origin) × 2 (low and high affectation) was carried out using the SWB as a dependent variable. An interaction effect was found between the province of origin and individual affectation, *F*(1) = 3.96, *p* < 0.05, showing that in the province of Ñuble those who presented greater affectation associated with the earthquake reported less SW. However, in the province of Concepción, the opposite effect was observed: the participants with the highest affectation reported the highest SW. In relation to community involvement, no significant results were found (see Figure 1).

### Intensity of Trauma, Social Sharing, Communal Appraisal, and Social Well-Being

**FIGURE 2:**
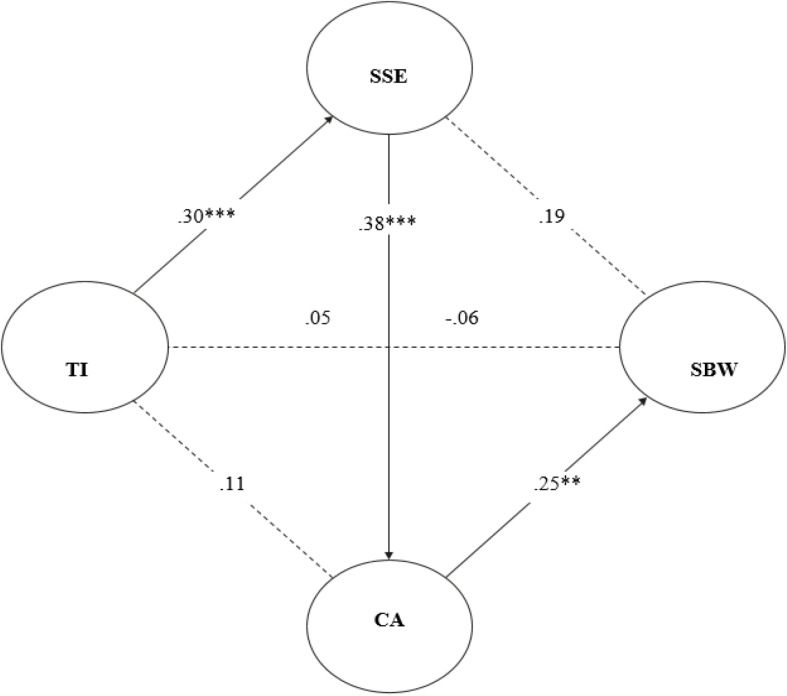
Relationship between TI and SBW mediated by SSE and CA.

A multiple mediation model was estimated to test the hypothesis of the relationship between trauma intensity and participation well-being through mediating variables of social sharing of emotions and communal appraisal (see Figure 2). The results confirm that there is no significant effect between the intensity of trauma and social well-being (*B* = −0.06, *t* = −0.69, *p* > 0.05). The direct effect of social sharing of emotions was significant with the intensity of the trauma (*B* = 0.30, *SE* = 0.03, *t* = 8.92, *p* = 0.000, 95% CI [0.23, 0.36]) and not with social well-being (*B* = 0.19, *SE* = 0.12, *t* = 0.53, *p* = 0.12, 95% CI [−0.05, 0.43]). Meanwhile, the direct effect of communal appraisal was not significant with the intensity of the trauma (*B* = 0.11, *SE* = 0.06, *t* = 1.73, *p* = 0.08, 95% CI [−0.01, 0.23]) and it was significant with social well-being (*B* = 0.25, *SE* = 0.06, *t* = 3.71, *p* = 0.0002, 95% CI [0.12, 0.39]). From the effects of the mediating variables, the non-significance between the intensity of the trauma and social well-being is maintained (*B* = 0.05, *SE* = 0.08, *t* = 0.57, *p* > 0.05, 95% CI [−0.12, 0.22]). Regarding the bootstraping test of indirect effects, the indirect effect that the mediator of communal appraisal contemplates is identified as significant (*B* = 0.04, Boot *SE* = 0.01, 95% CI [0.01, 0.08]).

## Conclusion

Based on the evidence that increased community action is a protective factor for mental health in the face of traumatic events (Gallagher et al., 2019), the study addressed how individuals and groups use community resources when dealing with the impacts of a natural disaster and, in this case, one of the most devastating earthquakes such as Chile’s 27F in 2010. The findings presented here support that people develop such resources as they experience the impacts and potential risks they face as a community, which are expressed in shared emotions and evaluations.

In this case, it is demonstrated that the intensity of trauma as an individual response experienced to the impact of the earthquake has a greater effect on the subsequent perception of social well-being through the processes of social sharing of emotions and communal appraisal. Although such processes have been specifically identified as collective coping strategies (Krum and Bandeira, 2008; Páez et al., 2013; Palacios and Barrios, 2013; Villagrán et al., 2014; Wlodarczyk et al., 2016a,b), it is proposed that they acquire relevance in different post-disaster phases and not only as a set of strategies involving different adaptive and maladaptive responses. On the contrary, according to the results of the multiple mediation model, the social expression of emotions and the communal appraisal would form a route so that the individual reactions could be projected later on in a social well-being. Together, the SSE and CA conform processes that would operate immediately, without waiting for the deployment of other strategies of collective confrontation. On the one hand, these processes would activate community organizing actions to address the demands of the disaster (e.g., rescue actions, provision of basic services, search for food; Pérez-Sales, 2004; Internacional Federation of Red Cross and Red Crescent Societies, 2008; Comité Permanente entre Organismos, 2009). On the other hand, the organization would make it possible to address other dangers commonly associated with natural disasters, to avoid looting or provide security actions in the face of rumors of threats from other groups (Quarantelli, 2008; Sanzana, 2010). Many of these actions would be activated in the face of difficulties in effectively coordinating government actions in natural disaster contexts (Bouckaert et al., 2010).

Although there is evidence affirming that sharing with others represents a negative experience that leads to the development of symptoms and/or disorders (Seery et al., 2008), the social sharing of emotions in a natural disaster context possess a different interpretation: the way people share their experience of distress and fear can, in itself, sustain their impact. According to Rimé (2009) sharing favors the recognition and communication of emotions in a context where they would not be judged or misunderstood and, on the contrary, would provide group cohesion, the strengthening of social bonds and would reveal the resistance of a community (Rimé et al., 2020).

In another sense, cognitive theories of emotion have suggested that they are not related to the event that gives rise to them, but rather to a cognitive assessment (Lazarus and Folkman, 1984). In this sense, appraisal involves not only evaluating an event, but also people’s behaviors, thoughts, or feelings (Manstead and Fischer, 2001). Hence, the latter assessment will guide us in the realization of our own evaluation of emotional events and, in a social context, the appraisal may occur when other people’s emotions trigger or modify our assessment of what is happening (Parkinson and Simons, 2009). At this point, the notion of the “communal or shared” implied by SSE and CA is relevant, as the findings show that the journey occurs when emotions are shared in an equally communal context of threat, strengthening social bonds, but that this process, by itself, does not lead to a resolution of the situations (Rimé et al., 2020). At this stage, an equally communal assessment (or what has been identified as a social appraisal) that would value aspects shared with others that can be expressed in a social identity (Tajfel et al., 1979) that would facilitate the identification of tasks and actions to be shared (Parkinson, 2019) to address such threats would operate. These results also provide guidance on the actions that can be achieved later in such a way that they facilitate the deployment of adaptive collective coping strategies that make it possible to adjust to the needs that arise in other post-disaster phases. There is abundant evidence that collective coping promotes better individual and collective responses to the demands of a disaster, however, its full deployment will take time and if the security, economic, political or psychosocial conditions are not in place, it may lead to unexpected or maladaptive responses.

Among the limitations of the study, it is reported that the evaluation that is carried out 4 years after the event that could generate responses associated with possible effects or memory biases after this time of the earthquake. Some findings suggest that, in the face of events such as violence, information from the environment could be positively analyzed to protect personal image (Bilbao et al., 2011). In addition, it is found that the Chilean population has a permanent exposure to the effects of earthquakes that have occurred after 27F (see Torres-Méndez et al., 2018) which could reduce the perception of damage and strengthen a positive social functioning. It may be more pertinent to conduct a longitudinal study that considers the medium- and long-term effects of experiences of this type.

Another aspect to highlight is the tendency to promote an irrefutable reading of the forms of organization and collective coping of the experiences associated with the earthquake, in line with informed findings (Villagrán et al., 2014; Wlodarczyk et al., 2016a,b). However, it would be of interest in future studies to consider the negative impacts of living a collective traumatic experience, for example, through the notion of psychosocial trauma formulated by Martín-Baró (1990) which accounts for the impact or “social wounding” from collective events such as violence. This would favor the expansion of the findings and exploring collective dynamics of distrust or resistance to change that persist. The vision of psychosocial trauma has been expanded to address scenarios such as the environmental damage caused by oil extraction in Ecuador (Sanandres and Otálora, 2015), the psychosocial effects on indigenous communities by the installation of dams in Mexico (Jiménez, 2014), and the impact of massive railroad accidents in Uruguay (Loarche, 2015), so it would be interesting to apply it to disaster contexts.

Despite this, it is worth considering that collective actions that could be developed among friends, family, neighbors and community members would encourage adaptive coping strategies at an immediate later stage, but do not necessarily entail medium- to long-term strategic actions aimed at disaster prevention and/or recovery (Sandoval et al., 2018). This limitation is associated with the wide variety of structural factors underlying risk, which cannot be driven by community actions (Maskrey, 2011). At this point, it is expected that State support will not only be oriented toward mitigating the effects of a disaster, but will also make it possible for reconstruction to consider community resources that make active participation possible (Krause et al., 2009; Caviedes, 2015).

## Data Availability Statement

The raw data supporting the conclusions of this article will be made available by the authors, without undue reservation.

## Ethics Statement

Ethical review and approval was not required for the study on human participants in accordance with the local legislation and institutional requirements. The patients/participants provided their written informed consent to participate in this study.

## Author Contributions

CR-V: data analysis, text writing, and translation. LV: data analysis and text writing. CA: text writing, translation, and formatting. FC: text writing and translation. JM: data collection and formatting. All authors contributed to the article and approved the submitted version.

## Conflict of Interest

The authors declare that the research was conducted in the absence of any commercial or financial relationships that could be construed as a potential conflict of interest.
